# Relationship between Accessory Renal Artery and Clinical Characteristics of Middle-Aged Patients with Primary Hypertension

**DOI:** 10.1155/2020/7109502

**Published:** 2020-04-08

**Authors:** Kai Kang, Yeshuo Ma, Chongfu Jia, Yunpeng Cheng, Yingchao Yang, Lixin Wang, Yinong Jiang, Yan Lu

**Affiliations:** ^1^Department of Cardiology, Institute of Cardiovascular Diseases, First Affiliated Hospital of Dalian Medical University, Dalian, Liaoning, China; ^2^Xiangya School of Pharmaceutical Sciences, Central South University, Changsha, Hunan, China; ^3^Department of Cardiology, The Third Xiangya Hospital of Central South University, Changsha, Hunan, China; ^4^Department of Cardiovascular Radiology, Institute of Cardiovascular Diseases, First Affiliated Hospital of Dalian Medical University, Dalian, Liaoning, China

## Abstract

**Objectives:**

The association between accessory renal artery (ARA) and hypertension remains not fully understood. We observed the association between ARA and clinical characteristics among middle-aged patients with primary hypertension.

**Methods:**

One hundred and sixty-two middle-aged (mean 39.82 ± 10.25 years, 58.0% male) patients with primary hypertension were enrolled, and patients underwent Computed Tomography Angiography (CTA) of renal arteries, ambulatory blood pressure monitor (ABPM), echocardiography, physical examination, and routine blood chemistry examinations. According to the CTA results, patients were divided into a non-ARA (*n* = 108) and ARA (*n* = 54) group. Direct renin concentration (DRC), plasma aldosterone concentration (PAC), ABPM, echocardiography, creatinine, and glomerular filtration rate were compared between the two groups.

**Results:**

DRC (mU/L) (11.21 (5.34, 20.87) vs. 18.24 (10.32, 33.59), *P*=0.002) was significantly higher in the ARA group than in the non-ARA group. However, PAC (ng/dL) (98.30 (67.30, 134.00) vs. 116.50 (78.80, 137.25), *P*=0.103) was similar between these two groups. ABPM (mmHg) results showed that daytime (146.75 ± 17.04/95.86 ± 11.39 vs. 155.50 ± 14.76/100.48 ± 10.69, *P* < 0.05), night time (133.44 ± 17.50/85.28 ± 12.80 vs. 139.81 ± 14.64/89.83 ± 11.21, *P* < 0.05), and 24 h blood pressure (143.95 ± 15.99/93.90 ± 11.78 vs. 152.07 ± 13.85/98.11 ± 10.36, *P* < 0.05) were significantly higher in the ARA group than in the non-ARA group. Accordingly, echocardiographic-derived posterior left ventricular wall thickness value was higher in the ARA group than in the non-ARA group.

**Conclusion:**

ARA is related to higher blood pressure and higher direct renin concentration in middle-aged patients with primary hypertension, and these patients deserve stricter blood pressure control. Our results provide important evidence for that ARA is a cause of hypertension and target organs damages.

## 1. Introduction

Hypertension is one of the major risk factors for various cardiovascular events, such as stroke, myocardial infarction, heart failure, and chronic kidney disease [1–3]. Unfortunately, the etiology and pathophysiology of hypertension have still not been fully elucidated [4, 5]. An understanding of the pathophysiology of hypertension is essential to develop effective therapeutic strategy that contributes to the prevention of cardiovascular events [6].

There are about 22–24% hypertensive patients having accessory renal arteries (ARAs) [7, 8]. ARAs are aberrant arterial branches originating directly from the aorta and serve a small portion of renal parenchyma [9, 10]. Commonly, the renal parenchyma served by ARA secretes more renin than the other parenchyma served by main renal artery as a result of smaller calibre and lower perfusion pressure of ARA [11, 12]. Excessive renin contributes to abnormally elevated blood pressure through activation of the renin-angiotensin system [13]. Previous studies have shown that ARA is associated with renin-dependent hypertension as well as resistant hypertension [11, 14]. Nevertheless, other researchers suggest that ARA is just a vascular anomaly and not a cause of hypertension [7, 15]. Therefore, it is still controversial whether ARA plays a role in the pathogenesis of hypertension and the potential pathogenic mechanism underlying it.

In the present study, we observed the association between ARA and clinical characteristics among middle-aged patients with primary hypertension who underwent complete pharmacological wash-out to elimination the influence of the medicines on the sympathetic nerve system and renin-angiotensin system.

## 2. Materials and Methods

### 2.1. Patients Selection and Design of the Study

Between June 2017 and January 2019, 162 patients with primary hypertension undergoing Computed Tomography Angiography (CTA) of renal arteries were enrolled in this study. All the patients were between 18 and 65 years of age and underwent adequately preparation before performing CTA, echocardiography, ambulatory blood pressure monitor (ABPM), physical examination, and routine blood chemistry examinations. Angiotensin-converting enzyme inhibitors, angiotensin II receptor blockers, *β*-blockers, dihydropyridine calcium blockers, and clonidine were withdrawn for at least 2 weeks; aldosterone receptor antagonists were withdrawn for at least 6 weeks; diuretic was withdrawn for at least 4 weeks. Patients were prescribed nondihydropyridine calcium blocker (diltiazem) and/or *α*-blockers (doxazosine and terazosin) to control blood pressure. According to the CTA results, the patients were divided into a non-ARA group (*n* = 108) and ARA group (*n* = 54), and the clinical characteristics of them were collected and summarized in Table 1. Patients with the following diseases were excluded from this study: secondary hypertension, white coat hypertension, diabetes mellitus, renal artery stenosis, infection, liver or kidney dysfunction, malignant tumor, malnutrition, pregnant women, and surgery within one year. This study was approved by the Ethics Committee of First Affiliated Hospital of Dalian Medical University, and written informed consent was obtained from all the patients.

### 2.2. Statistical Analysis

Data were analyzed by using the SPSS software (version 20, IBM Corporation, Armonk, NY, USA). The Kolmogorov–Smirnov test was used to determine whether the data were normally distributed. Continuous variables with normal distribution were expressed as mean ± SD and compared by the Independent-Sample *t*-Test. Continuous variables with nonnormal distribution were expressed as median (25^th^ to 75^th^ percentile) and compared by the nonparametric *U* test. Categorical variables were expressed as percentage and compared by the chi-square test. *P* < 0.05 was defined to be statistically significant.

## 3. Results

### 3.1. Patient Population

There were 162 patients with primary hypertension were enrolled in this study. According to the CTA results, there were 108 patients without ARA and 54 patients with ARA. The baseline characteristics of them showed no significant differences (Table 1).

### 3.2. Increased Direct Renin Concentration (DRC) in Patients with ARA

Firstly, we estimated DRC and plasma aldosterone concentration (PAC) of these patients. DRC was significantly increased in the ARA group compared with that of the non-ARA group (Table 2). Unexpectedly, PAC was only slightly upregulated in the ARA group (Table 2).

### 3.3. Higher Blood Pressure Measured by ABPM of Patients with ARA

Secondly, we examined whether there was difference in the level of blood pressure between these patients. The results of ABPM showed that both SBP (systolic blood pressure) and DBP (diastolic blood pressure) in day/night/24 h in the ARA group were higher than those of the non-ARA group (Table 3). These results suggested that the patients with ARA had higher blood pressure and should obtain stricter blood pressure control.

### 3.4. Patients with ARA Had Severe Target Organs Damages

Then, we assessed whether patients with ARA had severe target organs damages. Although interventricular septal thickness was mildly increased, there was a significant increase in left ventricular posterior wall thickness in the ARA group as measured by echocardiography (Table 4). It suggested that the geometric changes of myocardium in the patients with ARA. Furthermore, there was a slight increase in creatinine and reduction in glomerular filtration rate (GFR) in the ARA group indicating that there was mild renal dysfunction of the patients with ARA (Table 5). These results suggested that patients with ARA had severer target organs damages.

## 4. Discussion

To investigate whether ARA was associated with hypertension accurately, middle-aged patients with primary hypertension without renal artery stenosis were enrolled in the present study. In addition, compared with previous study, patients in our study underwent complete pharmacological wash-out to elimination the influence of the medicines on the sympathetic nerve system and renin-angiotensin system. Therefore, the results of the present study provided stronger evidences for the relationship between ARA and clinical characteristics among middle-aged patients with primary hypertension.

The relationship between ARA and pathogenesis of hypertension was firstly suggested by the observational study conducted by Kuczera et al. in 2009 [16]. ARA is commonly longer and narrower than the main renal artery with lower perfusion pressure and higher resistance across the artery [10]. The renal parenchyma served by ARA secretes more renin than that served by main renal artery [11]. In turn, increased renin results in the activation of the renin-angiotensin system that contributes to the pathogenesis of hypertension [13]. In our study, patients with ARA had higher blood pressure with significantly increased DRC compared with those of patients without ARA, which were consistent with previous research [11]. Furthermore, we also examined PAC among these patients at the same time. Unexpectedly, PAC was only slightly increased in the patients with ARA. The main reason for this maybe the high-salt diet of people from North China that inhibits the secretion of PAC [17, 18]. These results suggested that this anatomical variant of renal artery was a cause of hypertension through excessive renin and activation of the renin-angiotensin system. Considering the higher blood pressure and activation of the renin-angiotensin system in the patients with ARA, we wondered whether these patients had severe target organs damages. As we speculated, our results showed that the patients with ARA had significantly geometric changes of the myocardium and mild renal dysfunction. Therefore, these results suggested that the patients with ARA should obtain stricter blood pressure control to prevent of severe cardiovascular events.

We recognize some potential limitations in our study. This is a single-center study with a small sample size of middle-aged patients with primary hypertension. Multicenter research with adequate sample size, consisting of hypertensive patients and normotensive population, is essential to acquire more accurate relationship between ARA and hypertension. Besides, the volume of blood flow in ARA and blood flow perfusion of kidney is not estimated in this study. Mechanical investigations are also important to uncover the role of ARA in elevated blood pressure in the future.

## 5. Conclusions

In conclusion, we confirmed that ARA contributed to higher blood pressure in patients with primary hypertension through excessive renin and activation of the renin-angiotensin system. In addition, we verified that patients with ARA had severe target organs damages. Therefore, these patients deserved stricter blood pressure control to prevent of severe cardiovascular events. Our results provided significant evidences for the aggressive treatment of hypertensive patients with ARA in daily clinical practice.

## Figures and Tables

**Table 1:** Baseline characteristics.

	Total (*n* = 162)	Non-ARA group (*n* = 108)	ARA group (*n* = 54)	*P* value non-ARA group vs. ARA group
Sex (M/F)	94/68	60/48	34/20	0.402
BMI (kg/m^2^)	26.96 ± 6.46	26.70 ± 7.32	27.49 ± 4.37	0.064
Ages (years)	39.82 ± 10.25	40.53 ± 10.38	38.48 ± 10.03	0.107
Duration of hypertension (years)	1.00 (0.20, 5.00)	1.00 (0.20, 4.00)	2.00 (0.58, 5.25)	0.158
Smoking (*N*, %)	46 (42.59%)	32 (29.63%)	14 (26.93%)	0.713
Family history (*N*, %)	84 (77.78%)	54 (50.00%)	30 (55.56%)	0.617
HR (beat/minute)	73.98 ± 8.59	73.15 ± 8.05	75.61 ± 9.56	0.655
T-Chol (mmol/L)	4.84 ± 0.95	4.86 ± 0.92	4.84 ± 1.00	0.881
TG (mmol/L)	1.56 (1.10, 2.18)	1.54 (1.11, 2.27)	1.62 (1.08, 2.17)	0.856
HDL-C (mmol/L)	1.21 ± 0.29	1.22 ± 0.30	1.21 ± 0.30	0.560
LDL-C (mmol/L)	2.69 ± 0.61	2.69 ± 0.57	2.71 ± 0.69	0.669

ARA, accessory renal artery; BMI, body mass index; HR, heart rate; T-Chol, total cholesterol; TG, triglycerides; HDL-C, high density lipoprotein cholesterol; LDL-C, low density lipoprotein cholesterol.

**Table 2:** DRC and PAC.

	Non-ARA group (*n* = 108)	ARA group (*n* = 54)	*P* value
DRC (mU/L)	11.21 (5.34, 20.87)	18.24 (10.32, 33.59)	0.002
PAC (ng/dL)	98.30 (67.30, 134.00)	116.50 (78.80, 137.25)	0.103

DRC, direct renin concentration; PAC, plasma aldosterone concentration; ARA, accessory renal artery.

**Table 3:** Ambulatory blood pressure monitor (ABPM).

	Non-ARA group (*n* = 108)	ARA group (*n* = 54)	*P* value
SBP (day) (mmHg)	146.75 ± 17.04	155.50 ± 14.76	0.002
DBP (day) (mmHg)	95.86 ± 11.39	100.48 ± 10.69	0.014
SBP (night) (mmHg)	133.44 ± 17.50	139.81 ± 14.64	0.023
DBP (night) (mmHg)	85.28 ± 12.80	89.83 ± 11.21	0.028
SBP (24 h) (mmHg)	143.95 ± 15.99	152.07 ± 13.85	0.002
DBP (24 h) (mmHg)	93.90 ± 11.78	98.11 ± 10.36	0.027

ARA, accessory renal artery; SBP, systolic blood pressure; DBP, diastolic blood pressure.

**Table 4:** Echocardiography.

	Non-ARA group (*n* = 108)	ARA group (*n* = 54)	*P* value
Left atrial diameter (mm)	34.88 ± 3.44	35.09 ± 3.60	0.191
LVID (mm)	46.60 ± 3.91	46.22 ± 3.52	0.941
PWT (mm)	9.68 ± 1.22	10.22 ± 1.03	0.002
IVST (mm)	10.27 ± 1.52	10.67 ± 1.37	0.110
E/e'	7.4 ± 2.3	7.2 ± 2.2	0.611

ARA, accessory renal artery; LVID, left ventricular diameter; PWT, left ventricular posterior wall thickness; IVST, interventricular septal thickness; E/e', early diastolic mitral orifice flow velocity/peak mitral annular velocity.

**Table 5:** Creatinine and GFR.

	Non-ARA group (*n* = 108)	ARA group (*n* = 54)	*P* value
Creatinine (*μ*mol/L)	63.32 ± 15.30	65.80 ± 13.94	0.318
GFR (mL/(min × 1.73 m^2^))	130.41 ± 26.97	127.40 ± 23.46	0.487

ARA, accessory renal artery; GFR, glomerular filtration rate.

## Data Availability

The data of the present study are not publicly available because they contain the personal information of the participants. They are only available from the corresponding author on reasonable request.

## References

[1] Lee M. H., Kim J.-G., Jeon S. B., Kang D.-W., Kwon S. U., Kim J. S. (2019). Pharmacologically induced hypertension therapy for acute stroke patients. *Journal of Stroke*.

[2] Nanchen D., Klingenberg R., Gencer B. (2019). Inflammation during acute coronary syndromes—risk of cardiovascular events and bleeding. *International Journal of Cardiology*.

[3] Yu Z., Rebholz C. M., Wong E. (2019). Association between hypertension and kidney function decline: the atherosclerosis risk in communities (ARIC) study. *American Journal of Kidney Diseases*.

[4] Chen K., Zhou M., Wang X., Li S., Yang D. (2019). The role of myokines and adipokines in hypertension and hypertension-related complications. *Hypertension Research*.

[5] Calvillo L., Gironacci M. M., Crotti L., Meroni P. L., Parati G. (2019). Neuroimmune crosstalk in the pathophysiology of hypertension. *Nature Reviews Cardiology*.

[6] Stewart M. H., Lavie C. J., Ventura H. O. (2019). Emerging therapy in hypertension. *Current Hypertension Reports*.

[7] Gupta A., Tello R. (2004). Accessory renal arteries are not related to hypertension risk: a review of MR angiography data. *American Journal of Roentgenology*.

[8] Lauder L., Ewen S., Tzafriri A. R. (2018). Renal artery anatomy assessed by quantitative analysis of selective renal angiography in 1,000 patients with hypertension. *EuroIntervention*.

[9] Sadeghi-Azandaryani M., Zimmermann H., Korten I. (2017). Altered renal functions in patients with occlusion of an accessory renal artery after endovascular stenting of an infrarenal aneurysm. *Journal of Vascular Surgery*.

[10] Jamkar A., Khan B., Joshi D. (2017). Anatomical study of renal and accessory renal arteries. *Saudi Journal of Kidney Diseases and Transplantation*.

[11] Glodny B., Cromme S., Reimer P., Lennarz M., Winde G., Vetter H. (2000). Hypertension associated with multiple renal arteries may be renin-dependent. *Journal of Hypertension*.

[12] Chan P. L., Tan F. H. S. (2018). Renin dependent hypertension caused by accessory renal arteries. *Clinical Hypertension*.

[13] Mowry F. E., Biancardi V. C. (2019). Neuroinflammation in hypertension: the renin-angiotensin system versus pro-resolution pathways. *Pharmacological Research*.

[14] VonAchen P., Hamann J., Houghland T. (2016). Accessory renal arteries: prevalence in resistant hypertension and an important role in nonresponse to radiofrequency renal denervation. *Cardiovascular Revascularization Medicine*.

[15] Davies E. R., Sutton D. (1965). Hypertension and multiple renal arteries. *The Lancet*.

[16] Kuczera P., Włoszczyńska E., Adamczak M., Pencak P., Chudek J., Więcek A. (2009). Frequency of renal artery stenosis and variants of renal vascularization in hypertensive patients: analysis of 1550 angiographies in one centre. *Journal of Human Hypertension*.

[17] Taylor A. H. M., Rankin A. J., McQuarrie E. P. (2018). Non-uniform relationship between salt status and aldosterone activity in patients with chronic kidney disease. *Clinical Science*.

[18] Nagata S., Kato J., Kuwasako K., Kitamura K. (2010). Plasma and tissue levels of proangiotensin-12 and components of the renin-angiotensin system (RAS) following low- or high-salt feeding in rats. *Peptides*.

